# Comparison of P wave indices between ultramarathon athletes and general population

**DOI:** 10.14814/phy2.70766

**Published:** 2026-02-16

**Authors:** Narawudt Prasertwitayakij, Pongsatorn Tungsuk, Sirianong Namwongprom, Teerapat Nantsupawat, Siriluck Gunaparn, Arintaya Phrommintikul, Wanwarang Wongcharoen

**Affiliations:** ^1^ Division of Cardiology, Department of Internal Medicine, Faculty of Medicine Chiang Mai University Chiang Mai Thailand; ^2^ Department of Radiology, Faculty of Medicine Chiang Mai University Chiang Mai Thailand

**Keywords:** atrial fibrillation, endurance exercise, P wave indices, ultramarathon

## Abstract

High‐intensity endurance exercise is linked to increased atrial fibrillation (AF) risk. P wave indices are established AF risk markers, but their role in ultramarathon athletes is unexplored. This study aimed to compare P wave indices between ultramarathon athletes and healthy controls. This cross‐sectional study enrolled 74 ultramarathon athletes and 38 age‐ and sex‐matched healthy volunteers (2:1 ratio). Athletes had completed ≥1 race of ≥100 km or ≥60 km in the past year. Participants with AF, atrial flutter, cardiovascular diseases, or structural heart abnormalities were excluded. Resting 12‐lead ECGs evaluated P wave indices: maximum P wave duration, P wave dispersion, P wave terminal force in lead V1 (PTFV1), and P wave amplitude in lead II (PWAII). The cohort's mean age was 44.8 ± 8.2 years, 70% male. Ultramarathon athletes had significantly higher maximum P wave duration (114.24 ± 7.95 ms vs. 105.76 ± 7.15 ms, *p* < 0.001), P wave dispersion (18.77 ± 5.75 ms vs. 7.87 ± 2.51 ms, *p* < 0.001), PTFV1 (5795.66 ± 3212.27 μV·ms vs. 2399.17 ± 1140.19 μV·ms, *p* < 0.001) and PWAII (0.16 ± 0.05 mV vs. 0.12 ± 0.04 mV). Abnormal P wave duration (>120 ms) and PTFV1 (≥4000 μV·ms) were significantly more prevalent in ultramarathon athletes (25.7% vs. 5.3%, *p* = 0.010; 63.5% vs. 5.3%, *p* < 0.001). Similar findings were observed across genders. Ultramarathon runners demonstrate significant atrial electrical remodeling, as evidenced by abnormal P wave indices, may have potential relevance to arrhythmia risk. Further longitudinal studies are warranted to assess clinical outcomes.

## 1 INTRODUCTION

Atrial fibrillation (AF) is the most common arrhythmia encountered in clinical practice and has significant implications for a patient's health, including stroke, heart failure, and mortality (Benjamin et al., 2019). Previous studies have indicated that low to moderate‐intensity exercises are associated with a reduced risk of AF and cardiovascular diseases (Valenzuela et al., 2023). However, vigorous exercise does not appear to provide the same protective effects against AF (Mozaffarian et al., 2008). Numerous cohort and retrospective studies have demonstrated a potential link between high‐intensity endurance exercise and an elevated risk of AF (Estes et al., 2017; La Gerche et al., 2022). The popularity of ultramarathon running has remarkably increased in recent years, attracting participants from diverse demographics, including older adults. To date, there has been limited research on the effects of ultramarathons on atrial structure and function. Notably, only one study has specifically investigated the acute effects of ultramarathon running on atrial function and the occurrence of supraventricular arrhythmias in master athletes (Cavigli et al., 2022). Despite this important contribution, there remains a significant gap in the literature regarding the long‐term risk of AF in this unique population. Understanding the long‐term risk is particularly relevant given that atrial remodeling and chronic stress from high‐intensity endurance activities may predispose athletes to AF over time.

P wave indices, such as P wave duration and dispersion, have been identified as surrogate markers of AF risk, offering a noninvasive approach to assess atrial structure and function (Aizawa et al., 2017; Hancock et al., 2009; Rasmussen et al., 2020). However, no studies have specifically explored P wave indices in ultramarathon athletes. Therefore, we aimed to address this gap by comparing P wave indices between ultramarathon athletes and individuals from the general population.

## 2 METHODS

### 2.1 Study design and participants

This cross‐sectional study enrolled ultramarathon athletes and age‐ and sex‐matched healthy volunteers between October 2023 and July 2024. Eligible ultramarathon athletes were those who had completed at least one race of 100 kilometers (km) or more, or at least one race of 60 km or more in the past year, and who participated in ultramarathon events held in Chiang Mai, Thailand, on October 7, 2023. Participants with a history of AF or atrial flutter, established cardiovascular diseases (including heart failure, coronary artery disease, or significant structural heart abnormalities) were excluded from the study. The control group consisted of age‐ and sex‐matched healthy volunteers who had never run more than 2 h and never achieved a running distance greater than 20 km.

The research protocol obtained ethical approval from the Faculty of Medicine, Chiang Mai University, Ethics Committee (approval number 345/2566, dated 18 September 2023). All procedures were conducted following the principles of the Declaration of Helsinki, and written informed consent was obtained from all participants. The study was registered in the Thai Clinical Trials Registry (TCTR) under registration number TCTR20230726001, with the first trial registration on 26 July 2023.

### 2.2 Data collection

The baseline characteristics, including age, body mass index, resting heart rate, blood pressure, underlying diseases, intensity of exercise (hours/week), cumulative hours, and cumulative distance of exercise, were recorded for both ultramarathon runners and healthy volunteers. Resting 12‐lead electrocardiograms (ECGs) were performed on both groups: the ultramarathon runners and the age‐ and sex‐matched general population. Among ultramarathon runners, the ECG was performed within 1 week before the ultramarathon race on 7th October 2023. Echocardiography was performed on all subjects to exclude significant structural heart disease.

All 12‐lead ECGs were collected using a conventional ECG machine (Edan SE‐1200, Shenzhen, China). The ECGs were calibrated to 10 mm/mV with a paper speed of 25 mm/s. Successfully acquired ECGs were printed as hard copies and stored as digital image files (PDF format). The data were exported from the ECG machine via a universal serial bus (USB) port to a USB flash drive without compression. ECG measurements were made using EP Caliper software (EP Studios, Version 2.5.1 (48)).

P wave indices measured in this study included maximum P wave duration, P wave dispersion, P wave terminal force in lead V1, and P wave amplitude in lead II. The maximum P wave duration was measured from the initiation of the P wave onset to the isoelectric line before the QRS complex in the lead with the longest P wave duration. P wave dispersion was measured as the difference between the maximal and minimal P wave duration. The P wave terminal force in lead V1 (PTFV1) was calculated by multiplying the duration (ms) and depth (μV) of the negative deflection of the P wave in lead V1. A P‐wave duration ≥120 ms and a P‐wave dispersion ≥40 ms were considered abnormal. An abnormal P‐wave terminal force in lead V1 was defined as >4000 μV·ms. Additionally, P wave amplitude was recorded, and the presence of P wave amplitude in lead II greater than 0.25 mV, indicating right atrial abnormality, was noted. Leads with ambiguous or low‐amplitude P‐waves were excluded from measurement.

### 2.3 Statistical analysis

#### 2.3.1 Sample size calculation

**FIGURE 1:**
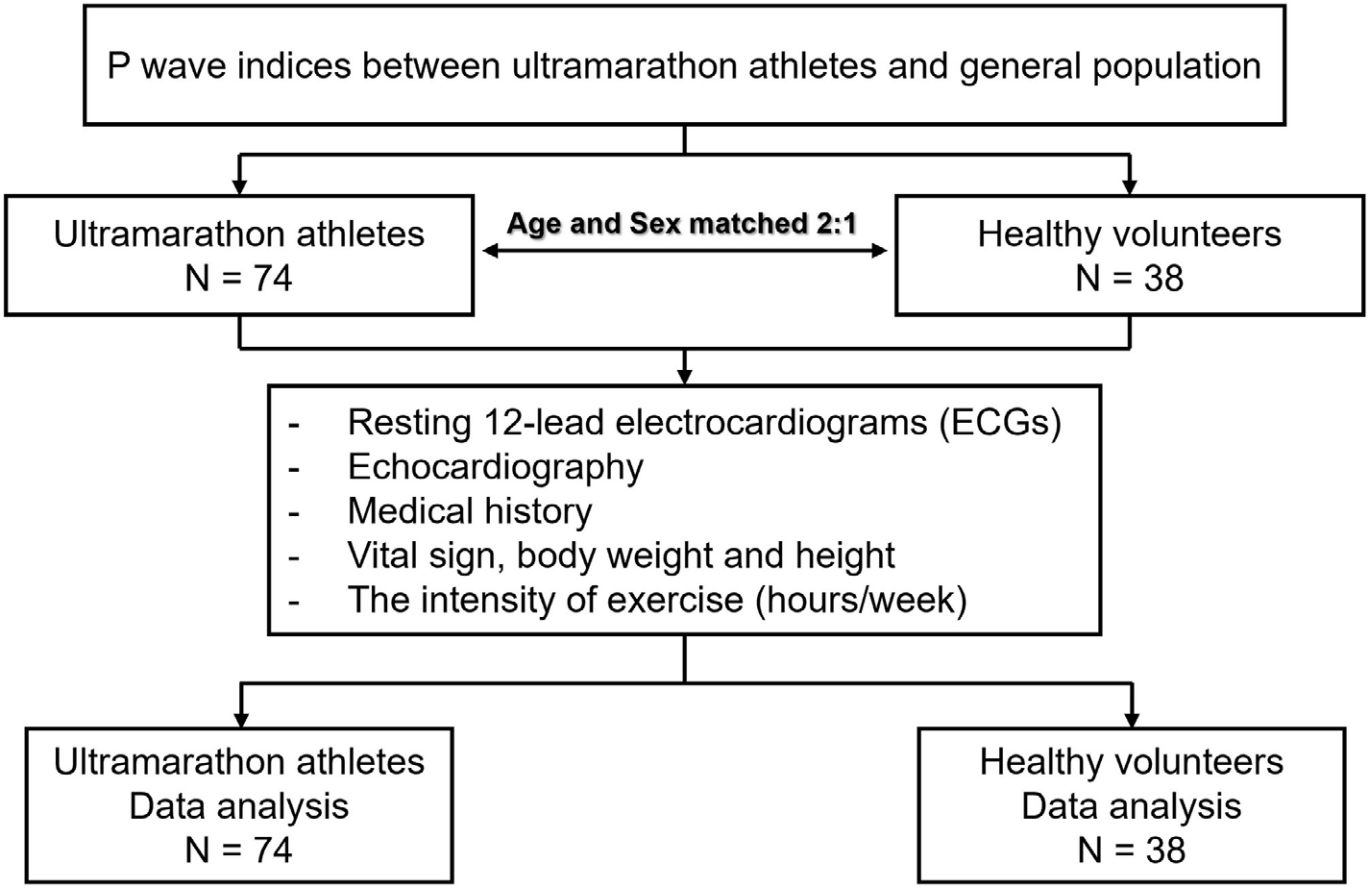
Study flow diagram.

A previous study showed that athletically fit adults had a longer P wave duration compared to athletically unfit adults (123 ± 9 ms vs. 96 ± 10 ms). (Dorey et al., 2019) Given an effect size of 2.79, power of 0.80, α of 0.05, and an adjusted group allocation of 2:1 (case: control), the minimum number of 52 subjects in the case group and 26 subjects in the control group were required. Because such a large effect size may overestimate the true between‐group difference, we increased our sample size and ultimately enrolled 74 cases and 38 controls to mitigate this potential bias (Figure 1).

#### 2.3.2 Data analysis

To compare numerical variables between groups, a *t*‐test was applied for values with a normal distribution, while the Mann–Whitney U test was used for those without a normal distribution. Proportions were analyzed using either the Chi‐square test or Fisher's exact test. Continuous variables are expressed as mean ± standard deviation (SD) or median with interquartile range (IQR), as appropriate. The difference of P wave parameters between groups is presented as mean (95% confidence interval). Whereas categorical variables are presented as percentages. Statistical significance was defined as a *p* value of less than 0.05. Data analysis was conducted using SPSS software, version 27 (IBM Corp., Armonk, NY, USA, https://www.ibm.com/products/spss‐statistics).

## 3 RESULTS

### 3.1 Baseline characteristics

A total of 74 ultramarathon runners met the inclusion criteria, and 38 individuals from the control population were enrolled in the study. Baseline characteristics and clinical data are shown in Table 1. The mean age of the ultramarathon runners was comparable to that of the control group. The majority of participants were male (70%). The mean body mass index was significantly lower in ultramarathon runners compared to the control group (22.44 ± 2.40 kg/m^2^ vs. 24.46 ± 3.15 kg/m^2^, *p* = 0.001). The mean systolic and diastolic blood pressures did not differ significantly between the two groups. Notably, the mean heart rate was significantly lower in ultramarathon runners than in controls (71.93 ± 12.26 vs. 82.37 ± 11.91 bpm, *p* < 0.001).

**TABLE 1:** Baseline characteristics.

Characteristic	Ultramarathon group	Control group	*p* Value
	(*N* = 74)	(*N* = 38)	
Age (years)	44.89 ± 7.52	44.76 ± 9.56	0.938
Male (%)	52 (70.30%)	26 (68.40%)	0.832
Height (cm)	167.04 ± 7.54	164.68 ± 8.02	0.128
Body weight (kg)	62.91 ± 10.09	66.74 ± 12.27	0.080
Body mass index (kg/m^2^)	22.44 ± 2.40	24.46 ± 3.15	0.001
Systolic blood pressure (mmHg)	133.54 ± 19.931	126.71 ± 14.67	0.065
Diastolic blood pressure (mmHg)	79.92 ± 12.01	76.87 ± 11.89	0.204
Heart rate (bpm)	71.93 ± 12.26	82.37 ± 11.91	<0.001
Duration of vigorous endurance exercise (years)	6.0 (4.0, 7.0)		
Hours of endurance exercise per week (hours)	70 (59, 129)		
Cumulative hours of endurance exercise (hours)	2355 (1569, 3647)		
Cumulative distance of running (km)	18,000 (10,728, 23,912)		

We demonstrated that the maximum P wave duration was significantly longer in the ultramarathon group compared to the control group (114.24 ± 7.95 ms vs. 105.76 ± 7.15 ms, *p* < 0.001). The proportion of participants with abnormal P wave duration (≥120 ms) was also significantly higher in the ultramarathon group than in the control group (25.7% vs. 5.3%, *p* = 0.010). In terms of P wave dispersion, the ultramarathon group showed markedly higher values compared to the control group (18.77 ± 5.75 ms vs. 7.87 ± 2.51 ms, *p* < 0.001). However, no participants in either group exhibited abnormal P wave dispersion (≥40 ms). A significant difference was observed in the P wave terminal force in lead V1, with ultramarathon runners demonstrating substantially higher values compared to the control group (5795.66 ± 3212.27 μV·ms vs. 2399.17 ± 1140.19 μV·ms, *p* < 0.001). Furthermore, the prevalence of abnormal P wave terminal force in lead V1 (≥4000 μV·ms) was significantly greater in the ultramarathon group compared to the control group (63.5% vs. 5.3%, *p* < 0.001). In addition, P wave amplitude in lead II was significantly greater in ultramarathon runners than the controls (Table 2).

**TABLE 2:** P wave indices compare ultramarathon and control groups.

P wave indices	Ultramarathon	Control	Mean difference (95% CI)	*p* Value
	(*N* = 74)	(*N* = 38)		
Maximum P wave duration (ms)	114.24 ± 7.95	105.76 ± 7.15	8.48 (5.54–11.52)	<0.001
Abnormal P wave duration (*n*, %)	19 (25.7%)	2 (5.3%)	N/A	0.010
P wave dispersion (mS)	18.77 ± 5.75	7.87 ± 2.51	10.90 (8.96–12.84)	<0.001
Abnormal P wave dispersion (*n*, %)	0 (0%)	0 (0%)	N/A	
P wave terminal force in V1 (μV·ms)	5795.66 ± 3212.27	2399.17 ± 1140.19	3396.49 (2328.98–4464.00)	<0.001
Abnormal P wave terminal force in V1 (*n*, %)	47 (63.5%)	2 (5.3%)	N/A	<0.001
P wave amplitude in lead II (mV)	0.16 ± 0.05	0.12 ± 0.04	0.45 (0.06–0.28)	<0.001
Abnormal P wave amplitude in lead II	2 (2.7%)	0 (0%)	N/A	0.548

Abbreviations: CI, confidence interval; N/A, not available; ms, milliseconds, mV, millivolts.

We further investigated the effect of gender on the disparity in P wave indices between the ultramarathon and control groups. Significant differences in P wave indices were observed between ultramarathon runners and controls in both males and females (Table 3).

**TABLE 3:** P wave indices compare ultramarathon and control groups stratified by gender.

P wave indices	Male			Female		
	Ultramarathon	Control	*p* Value	Ultramarathon	Control	*p* Value
	*N* = 52	*N* = 26		*N* = 22	*N* = 12	
Maximum P wave duration (ms)	115.09 ± 7.74	106.81 ± 7.64	<0.001	110.32 ± 7.17	103.50 ± 5.58	0.005
P wave dispersion (ms)	18.67 ± 5.59	7.39 ± 2.42	<0.001	19.00 ± 6.23	8.92 ± 2.47	<0.001
P wave terminal force in V1 (μV·ms)	5976.09 ± 3467.43	2447.72 ± 1128.75	<0.001	5369.19 ± 2529.76	2293.96 ± 1208.11	<0.001
P wave amplitude in lead II (mV)	0.17 ± 0.05	0.12 ± 0.04	<0.001	0.16 ± 0.05	0.11 ± 0.04	0.010

Among the 74 ultramarathon runners enrolled in our study, the median cumulative running distance was 18,000 km (IQR 10,728–23,912 km). To evaluate the dose–response effect of endurance exercise on P wave indices, we compared the indices between ultramarathon runners with a cumulative running distance of ≤18,000 km and those with >18,000 km. The results showed no statistically significant differences between the two groups across all examined parameters (Table 4). Similarly, there were no significant differences in P wave indices between ultramarathon runners with cumulative endurance exercise hours of ≤2000 h and those with >2000 h.

**TABLE 4:** Comparison of P wave indices between cumulative distance (≤ 18,000 km and >18,000 km) and cumulative hours (≤2000 km and >2000 km) among ultramarathon runners.

P wave indices parameter	Cumulative distance (≤18,000 km)	Cumulative distance (>18,000 km)	*p* Value	Cumulative hours (≤2000 km)	Cumulative hours (>2000 km)	*p* Value
Maximum P wave duration (ms)	114.07 ± 8.04	114.73 ± 8.34	0.764	115.04 ± 8.00	113.88 ± 8.37	0.590
P wave dispersion (ms)	18.10 ± 6.22	17.89 ± 4.16	0.881	19.08 ± 6.06	17.41 ± 4.59	0.261
P wave terminal force in V1 (μV·ms)	5508.73 ± 3318.18	6279.71 ± 3523.28	0.405	5301.20 ± 3560.28	6091.94 ± 3282.68	0.394
P wave amplitude in lead II (mV)	0.17 ± 0.48	0.17 ± 0.46	0.822	0.16 ± 0.04	0.17 ± 0.05	0.689

To assess the presence of a dose–response relationship between exercise volume and changes in P‐wave indices, we performed further correlation analyses between cumulative running distance and running hours with each P‐wave index among ultramarathon runners (Table 5). Notably, we found that neither cumulative running distance nor running duration was significantly correlated with any P‐wave indices.

**TABLE 5:** Correlation analysis of cumulative endurance exposure with P wave indices and echocardiographic parameters.

Parameters	Correlation analysis			
	Cumulative distance		Cumulative hours	
	Pearson correlation	*p* Value	Pearson correlation	*p* Value
P wave parameters				
Maximum P wave duration (ms)	0.034	0.801	−0.161	0.228
P wave dispersion (ms)	−0.006	0.964	−0.077	0.564
P wave terminal force in V1 (μV·ms)	−0.001	0.994	−0.057	0.67
P wave amplitude in lead II (mV)	−0.103	0.443	−0.067	0.617
Echocardiography parameters				
E/e′ ratio	−0.015	0.917	0.072	0.614
Left atrial volume index (mL/m^2^)	0.294	0.047*	0.233	0.120
Left ventricular mass index (g/m^2^)	0.104	0.465	−0.100	0.481
TRV max (cm/sec)	−0.072	0.729	0.012	0.952

Abbreviations: E/e′, E peak velocity/septal e′ velocity; TRV, tricuspid regurgitation velocity.

* *p* < 0.05.

In an analysis of echocardiographic parameters, both the ultramarathon and control groups demonstrated normal left ventricular ejection fraction (LVEF), with no statistically significant difference between them (61.39 ± 6.36% vs. 59.19 ± 6.31%, *p* = 0.116). Similarly, mean left atrial (LA) diameter remained comparable between the two groups (3.24 ± 0.40 vs. 3.10 ± 0.43 cm, *p* = 0.121). Regarding diastolic function, no significant differences were observed in septal e′ velocity (9.86 ± 2.01 vs. 9.56 ± 2.40 cm/s, *p* = 0.551) or the E/e′ ratio (7.22 ± 2.00 vs. 6.93 ± 1.57, *p* = 0.440).

In contrast, ultramarathoners exhibited evidence of structural and hemodynamic remodeling. The mean LA volume index (LAVI) was significantly higher in the ultramarathon group compared to controls (25.5 ± 8.62 vs. 22.5 ± 7.44 mL/m^2^, *p* = 0.049). This was accompanied by a significantly greater LV mass index (88.90 ± 19.47 vs. 79.74 ± 15.06 g/m^2^, *p* = 0.013) and a higher peak tricuspid regurgitation velocity (TRV max: 213.21 ± 25.88 vs. 188.38 ± 26.54 cm/s, *p* = 0.008).

Further evaluation of dose–response relationships between cumulative endurance exposure and echocardiographic parameters of diastolic function is presented in Table 5. We found no significant correlations between cumulative endurance exposure and LV mass index, E/e′, or TRV. Notably, cumulative running distance was significantly associated with LAVI, although the correlation was weak (*r* = 0.294, *p* = 0.049).

## 4 DISCUSSION

The association between endurance exercise and AF has been well‐documented, with numerous studies demonstrating that endurance athletes have a higher risk of AF compared to the general population (Estes et al., 2017; Guasch et al., 2013; Mont et al., 2009; Mozaffarian et al., 2008; Woodward et al., 2015). The repetitive atrial strain caused by prolonged and intensive physical activity is thought to lead to structural and electrical remodeling of the atria, increasing susceptibility to arrhythmias such as AF (Dorey et al., 2019; Guasch et al., 2013; Guasch & Mont, 2017). While this relationship is established for endurance sports like cycling and marathons, ultramarathon running represents a unique form of endurance activity due to its extreme duration and intensity, often lasting several hours to days. This distinctive nature of ultramarathon running raises questions about its specific impact on atrial health.

The P wave indices, including P wave duration, P wave dispersion, and P wave terminal force in V1 are noninvasive ECG markers that reflect atrial conduction heterogeneity and can serve as surrogate markers of AF risk (Alexander et al., 2017; Nielsen et al., 2015; Rasmussen et al., 2020). Despite their potential utility, there has been a notable lack of studies exploring P wave indices in ultramarathon runners. Identifying abnormal P wave indices in this population could help pinpoint athletes at higher risk for developing AF, allowing for early interventions and tailored training modifications.

In our study, we demonstrated that ultramarathon runners had a significantly higher prevalence of abnormal P wave indices compared to age‐ and sex‐matched controls from the general population. Although the absolute values of P wave dispersion did not exceed conventional diagnostic cutoffs, the consistently higher dispersion observed in ultramarathon runners may still reflect a continuum of atrial adaptation rather than a binary normal–abnormal distinction. Prior electrophysiological studies indicate that P wave dispersion operates on a continuous scale, where incremental increases—even within the “normal” range—can signify greater atrial conduction inhomogeneity and heightened atrial vulnerability. In this context, the sub‐threshold elevations identified in our cohort may represent early electrical remodeling, with abnormal P wave indices serving as a potential surrogate marker for arrhythmia risk. While the prognostic implications cannot be determined from a cross‐sectional design, these subtle differences underscore the need for longitudinal monitoring to clarify whether such early conduction variability predicts future atrial arrhythmias in ultramarathon runners.

Our findings differ from the nonconventional electrocardiography study, using signal‐averaged electrocardiographs for filtered P‐wave duration and atrial late potentials, with no difference noted after 10 months of 5–6 h/week compared to the stretch/balance exercise control group (McNamara et al., 2019). This discrepancy may be explained not only by differences in electrocardiographic methodology but also by differences in study populations. In particular, our study focused exclusively on experienced ultramarathon runners, who had a median cumulative endurance running exposure of approximately 2000 h.

In addition, our findings were in contrast with those reported in the prior studies, (Mohanty et al., 2016) which demonstrated sex‐specific association with AF risk. Specifically, the prior meta‐analysis observed that male athletes were more likely to exhibit a higher risk of AF compared to their female counterparts, highlighting a potential gender‐related susceptibility to atrial remodeling associated with endurance exercise. In contrast, our study did not identify significant differences in P wave indices between male and female ultramarathon runners. Both sexes exhibited similar P wave characteristics when compared to age‐ and sex‐matched controls. This indicates that ultramarathon running may induce comparable atrial electrical remodeling in both male and female athletes. The discrepancy between our findings and those of prior studies may be attributed to differences in the populations studied and the nature of endurance activities. The prior study examined a broader cohort of endurance athletes participating in various sports, while our study focused specifically on ultramarathon runners, a subgroup exposed to extreme and prolonged endurance stress. The absence of gender‐specific differences in our study could suggest that the extreme and sustained nature of ultramarathon training and competition overrides any gender‐related predispositions to atrial remodeling observed in other endurance sports.

In our study, LAVI was larger in ultramarathon runners than in the control group, a finding consistent with previous studies in healthy individuals and in populations with abnormal left ventricular filling pressures following exercise (Kyrouac et al., 2025; McNamara et al., 2019). These observations support the concept of training‐induced atrial remodeling associated with sustained endurance exercise (Kyrouac et al., 2025). Moreover, persistence of cardiac remodeling has been reported in former elite athletes even after prolonged deconditioning, raising the hypothesis that age‐related ventricular stiffness or irreversible atrial fibrosis may contribute to sustained atrial enlargement (Aaroee et al., 2025).

Previous studies have demonstrated a dose–response relationship between physical activity and the risk of AF in the general population (Calvo et al., 2016), suggesting that increasing levels of physical activity are associated with a higher risk of AF and highlighting the cumulative effects of endurance exercise on atrial remodeling. In the present study, we did not observe a significant correlation between cumulative endurance exercise and P‐wave indices. However, a positive association between training volume and left atrial volume index was identified. This finding is consistent with prior reports supporting a dose–response relationship between exercise volume and atrial structural remodeling. The absence of a corresponding association with P‐wave indices may reflect the lower sensitivity of surface ECG markers compared with echocardiographic measures such as LAVI for detecting exercise‐related atrial remodeling.

Our results underscore the importance of incorporating routine P wave analysis into cardiovascular evaluations for endurance athletes, particularly those engaged in ultramarathon running. Future longitudinal studies to confirm whether these abnormal P wave indices as a surrogate finding of atrial remodeling in ultramarathon athletes are warranted.

Our study has several limitations. The first limitation is the selection of a control group with minimal endurance exposure. While this approach allowed us to compare ultramarathon runners with individuals at the low end of the endurance‐training spectrum, it also creates a pronounced contrast that may magnify the observed differences in atrial electrical and structural indices. As a result, the generalizability of our findings to moderately trained or recreational endurance runners is uncertain. These intermediate groups may exhibit subtler or graded patterns of atrial remodeling that fall between the two extremes represented in our study. Future studies incorporating a broader range of endurance‐training volumes—including recreational, competitive, and elite athletes—are warranted to determine whether ultramarathon‐associated atrial changes occur along a continuum or reflect a threshold effect of extreme, sustained training loads. Secondly, this study lacks longitudinal follow‐up and clinical outcome data. Given the cross‐sectional design, we are unable to determine the temporal evolution, persistence, or reversibility of the observed P‐wave indices in response to changes in training volume or detraining. Prior studies in former elite athletes suggest that exercise‐induced atrial remodeling may be irreversible with reduced training load, whereas prolonged exposure to high‐volume endurance exercise may lead to more sustained structural and electrophysiological adaptations in a subset of athletes (Aaroee et al., 2025). In this context, the P‐wave indices evaluated in our study should be interpreted as subclinical electrophysiological markers rather than direct surrogates of atrial arrhythmia risk or prognosis. Future prospective studies incorporating longitudinal ECG assessments, detraining periods, and clinical outcomes such as incident AF are needed to clarify the clinical significance and prognostic implications of these findings.

Thirdly, the power calculation relied on a previously reported effect size of 2.79, which is unusually large and may overestimate the expected group difference. Although this could limit the precision of the initial power estimate, our final sample size substantially exceeded the calculated minimum, reducing the likelihood that the study was underpowered despite the optimistic effect‐size assumption. Based on the observed data in our cohort (mean P‐wave duration 114.24 ± 7.95 vs. 105.76 ± 7.15), the corresponding effect size was 1.07. A post hoc power analysis using these observed values indicated a statistical power of >0.99 at α = 0.05. This confirms that the study remained adequately powered despite the conservative nature of our initial assumptions.

A further limitation relates to the heterogeneity of training exposure within the ultramarathon cohort. Although we incorporated available indicators of endurance load—including cumulative running distance and cumulative training hours—to examine continuous dose–response relationships, more granular aspects of training history (such as weekly mileage patterns, number and timing of ultramarathon events, or periodization strategies) were not collected. As a result, our ability to fully characterize within‐group variability is limited, and some nuances of training‐related adaptation may not have been captured. Future studies that include detailed training logs or stratified endurance profiles will be better positioned to clarify how differing training histories contribute to electrical and structural atrial remodeling in ultramarathon athletes. Lastly, the use of a single resting 12‐lead ECG recorded before the competition may not accurately reflect chronic or long‐term changes in atrial electrical activity.

## 5 CONCLUSION

Ultramarathon runners demonstrate significant atrial electrical remodeling, as evidenced by abnormal P wave indices, and may have potential relevance to arrhythmia risk. Further longitudinal studies are warranted to assess clinical outcomes.

## AUTHOR CONTRIBUTIONS

NP had the lead role in conceptualization, drafting the original manuscript, and formal analysis, and contributed equally to the review and editing process. PT and SN contributed to the development of the study proposal, methodology, and data curation. TN contributed to the methodology and led the data curation efforts. SG oversaw project administration and provided support for data curation. AP contributed to the review and editing of the manuscript. WW provided support for the conceptualization and drafting of the original manuscript and contributed equally to the review and editing process. All authors agreed to be accountable for all aspects of the work in ensuring that questions related to the accuracy or integrity of any part of the work are appropriately investigated and resolved.

## FUNDING INFORMATION

This work was supported by the Faculty of Medicine Endowment Fund for Medical Research (Approval ID 088/2024), Faculty of Medicine, Chiang Mai University, Chiang Mai, Thailand. The funding source had no role in the design and conduct of the study; the collection, management, analysis, or interpretation of the data; the preparation, review, or approval of the manuscript; or the decision to submit the manuscript for publication.

## CONFLICT OF INTEREST STATEMENT

All the authors declare that they have no conflict of interests.

## ETICS STATEMENT

This study was granted by the Faculty of Medicine, Chiang Mai University, Ethics Committee (approval number 345/2566, September 18, 2023). The study adhered to the principles outlined in the Declaration of Helsinki. All participants provided written informed consent. This study was registered with the Thai Clinical Trials Registry (TCTR20230726001, registered July 26, 2023).

## DECLARATION OF GENERATIVE AI IN SCIENTIFIC WRITING

Generative AI technologies were not used in the conception, writing, or revision of this manuscript.

## Data Availability

The datasets generated during and/or analyzed during the current study are not publicly available due to the institution's regulations but are available from the corresponding author via email (wanwarang.w@cmu.ac.th) upon reasonable request.
